# Osteoporosis under psychological stress: mechanisms and therapeutics

**DOI:** 10.1093/lifemedi/lnae009

**Published:** 2024-03-07

**Authors:** Hao-Kun Xu, Jie-Xi Liu, Ze-Kai Zhou, Chen-Xi Zheng, Bing-Dong Sui, Yuan Yuan, Liang Kong, Yan Jin, Ji Chen

**Affiliations:** State Key Laboratory of Oral & Maxillofacial Reconstruction and Regeneration, National Clinical Research Center for Oral Diseases, Shaanxi International Joint Research Center for Oral Diseases, Center for Tissue Engineering, School of Stomatology, The Fourth Military Medical University, Xi’an 710032, China; Department of Oral Anatomy and Physiology, School of Stomatology, The Fourth Military Medical University, Xi’an 710032, China; State Key Laboratory of Oral & Maxillofacial Reconstruction and Regeneration, National Clinical Research Center for Oral Diseases, Shaanxi International Joint Research Center for Oral Diseases, Center for Tissue Engineering, School of Stomatology, The Fourth Military Medical University, Xi’an 710032, China; State Key Laboratory of Oral & Maxillofacial Reconstruction and Regeneration, National Clinical Research Center for Oral Diseases, Shaanxi International Joint Research Center for Oral Diseases, Center for Tissue Engineering, School of Stomatology, The Fourth Military Medical University, Xi’an 710032, China; School of Basic Medicine, The Fourth Military Medical University, Xi’an 710032, China; State Key Laboratory of Oral & Maxillofacial Reconstruction and Regeneration, National Clinical Research Center for Oral Diseases, Shaanxi International Joint Research Center for Oral Diseases, Center for Tissue Engineering, School of Stomatology, The Fourth Military Medical University, Xi’an 710032, China; State Key Laboratory of Oral & Maxillofacial Reconstruction and Regeneration, National Clinical Research Center for Oral Diseases, Shaanxi International Joint Research Center for Oral Diseases, Center for Tissue Engineering, School of Stomatology, The Fourth Military Medical University, Xi’an 710032, China; State Key Laboratory of Oral & Maxillofacial Reconstruction and Regeneration, National Clinical Research Center for Oral Diseases, Shaanxi International Joint Research Center for Oral Diseases, Center for Tissue Engineering, School of Stomatology, The Fourth Military Medical University, Xi’an 710032, China; Exercise Immunology Center, Wuhan Sports University, Wuhan 430079, China; Department of Oral and Maxillofacial Surgery, School of Stomatology, The Fourth Military Medical University, Xi’an 710032, China; State Key Laboratory of Oral & Maxillofacial Reconstruction and Regeneration, National Clinical Research Center for Oral Diseases, Shaanxi International Joint Research Center for Oral Diseases, Center for Tissue Engineering, School of Stomatology, The Fourth Military Medical University, Xi’an 710032, China; State Key Laboratory of Oral & Maxillofacial Reconstruction and Regeneration, National Clinical Research Center for Oral Diseases, Shaanxi International Joint Research Center for Oral Diseases, Center for Tissue Engineering, School of Stomatology, The Fourth Military Medical University, Xi’an 710032, China; Department of Oral Implantology, School of Stomatology, The Fourth Military Medical University, Xi’an 710032, China

**Keywords:** psychological stress, osteoporosis, sympathetic nervous system, type H vessels, extracellular vesicles

## Abstract

Psychological stress has been associated with the onset of several diseases, including osteoporosis. However, the underlying pathogenic mechanism remains unknown, and effective therapeutic strategies are still unavailable. Growing evidence suggests that the sympathetic nervous system regulates bone homeostasis and vascular function under psychological stress, as well as the coupling of osteogenesis and angiogenesis in bone development, remodeling, and regeneration. Furthermore, extracellular vesicles (EVs), particularly mesenchymal stem cell extracellular vesicles (MSC–EVs), have emerged as prospecting therapies for stimulating angiogenesis and bone regeneration. We summarize the role of sympathetic regulation in bone homeostasis and vascular function in response to psychological stress and emphasize the relationship between vessels and bone. Finally, we suggest using MSC–EVs as a promising therapeutic method for treating osteoporosis in psychological stress.

## Introduction

Psychological stress is a state of tension characterized by physiological, emotional, and behavioral changes due to adversity, perceived stress, or threat [1–3]. People are experiencing significantly increased psychological pressure and stress caused by negative life events, which seriously impact physical health as the pace of life and work continues to accelerate [4]. Under normal circumstances, the stress response system has robust basic activity and response capabilities, critical for happiness, task achievement, and appropriate social interaction. However, the excessive or insufficient activity of this system impairs development and growth, resulting in various behavioral changes and pathological conditions [5]. Increasing evidence suggests that chronic psychological stress affects brain function and disrupts the balance of the stress system, resulting in inflammatory diseases, cardiovascular diseases, obesity, diabetes, atherosclerosis, cancer, neurodegenerative diseases, and osteoporosis [6–12].

Osteoporosis is a systemic skeletal disease that causes reduced bone mass and deterioration of bone tissue microstructure, increasing the risk of fractures [13]. Osteoporosis affected over 12.6% of non-institutionalized Americans aged 50 and over in 2017–2018 [14]. In 2017, 20 million people aged 50 or older in Europe had osteoporosis, with 2.7 million suffering osteoporotic fractures, resulting in a total treatment cost of 37.5 million euros and a considerable economic burden on society [15]. An increasing body of evidence indicates a substantial correlation between chronic psychological stress and bone loss [16–18], with studies revealing a positive relationship between the risk of osteoporotic fractures and psychological stress [19]. Furthermore, individuals diagnosed with post-traumatic stress disorder (PTSD) are more likely to develop osteoporosis [20], and long-term chronic psychological stress leads to depression, which is related to reduced bone mineral density (BMD) compared to non-depressed individuals [21].

Although earlier research has suggested a tight relationship between osteoporosis and psychological stress, the underlying mechanism and effective treatment techniques and approaches for osteoporosis under chronic psychological stress remain unknown and unestablished. We summarize the correlation between psychological stress and osteoporosis and highlight the function of the sympathetic nervous system (SNS) in regulating bone homeostasis. We also discuss the role of vasculature in bone development, remodeling, and regeneration, along with the effects of SNS on vascular regulation in bone. Furthermore, we focus on emerging therapeutics of extracellular vesicles (EVs), particularly mesenchymal stem cell-derived EV (MSC–EVs). We detail the impacts and mechanisms underpinning the use of MSC–EVs in vascular regulation and bone regeneration and propose the MSC–EV engineering strategy to enhance their efficiency and achieve more precise targeting.

## Psychological stress and osteoporosis

### Response mechanisms and sympathetic nervous system effects under psychological stress

**Figure 1.**
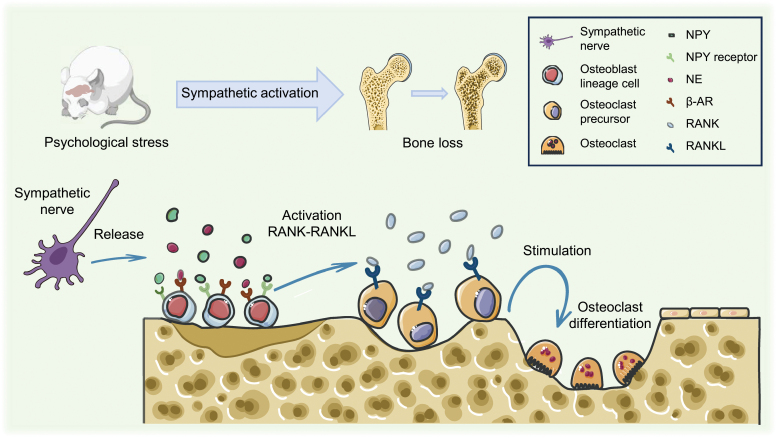
**Psychological stress-induced sympathoexcitation leads to bone loss.**Activation of sympathetic nerves triggers the release of NE and NPY, which bind to osteoblast β-AR and NPY receptors, initiating the RANK-RANKL pathway and driving osteoclast differentiation, ultimately resulting in bone loss. NE, norepinephrine; β-AR, beta-adrenergic receptor; NPY, neuropeptide Y.

The neuroendocrine response to stress causes numerous metabolic and functional changes in multiple organs and cells to maintain individual homeostasis in the face of possible dangers. The hypothalamus-pituitary-adrenal (HPA) axis and the sympathetic-adrenal medullary (SAM) axis are the two most important neuroendocrine response axes [22]. The HPA axis is activated when the hypothalamus secretes corticotropin-releasing hormone (CRH), stimulating adrenocorticotropic hormone (ACTH) release from the pituitary. As a result, glucocorticoid (GC) production and secretion from the adrenal cortex are regulated. GCs play crucial roles in reacting to harmful stimuli, maintaining blood pressure and blood sugar levels, mobilizing lipids, fighting cell damage, and regulating inflammatory reactions. Sympathetic activation increases, and norepinephrine (NE) is released from sympathetic nerve terminals when SAM axis is activated (Fig. 1). This activates the adrenal medulla to rapidly generate large amounts of catecholamines, mobilizing and regulating respiration, metabolism, and blood circulation, resulting in a state of alertness in the body and ensuring that critical organs can meet stress demands [23–26].

The SNS is widely distributed, with postganglionic fibers from neurons in the paravertebral ganglia innervating the pupils, heart, respiratory system, blood vessels, facial and jaw muscles, trunk, and exocrine glands of the limbs. At the same time, postganglionic fibers from neurons in the prevertebral ganglia innervate the organs in the abdomen, pelvis, and perineum [27]. NE is the main SNS neurotransmitter, transmitting signals through adrenergic receptors (ARs), which are classified into α and β types and are both G protein-coupled receptors [28]. Dysfunction of the SNS is closely related to the onset and progression of many diseases, such as peripheral neuropathy, congestive heart failure, hypertension, diabetes, and immune dysfunction [29–33].

### Regulation of bone homeostasis under psychological stress

Bones are critical organs that maintain posture and mobility, protect vital organs, and serve as crucial sites for hematopoiesis and mineral storage, in addition to playing an essential role in endocrine regulation and homeostasis [34, 35]. In the early 21st century, cross-sectional investigations on bone mass, body weight, and gonadal function have revealed new insights into how the nervous system regulates bone remodeling. Leptin was discovered in 2002 to alter SNS tone by acting on the hypothalamus, resulting in the release of NE into the local microenvironment and the activation of β-adrenergic receptors (β-ARs) produced by osteoblasts to modulate bone mass [36]. Additionally, neuropeptide U also regulates bone mass by signal processing through the SNS [37]. As research on the local microenvironment of bone progresses, the crucial regulatory role of SNS in bone metabolism has gradually emerged. The SNS is widely distributed in the periosteum and bone, densely distributed in the growth plate and metaphysis of long bones, and runs parallel to blood vessels. ARs and neuropeptide receptors have also been detected in osteoblasts and osteoclasts [38–40]. These findings imply that the SNS regulates the role of bone tissue.

Regulation of bone homeostasis is critical for adapting to changes in the physiology and external environment while maintaining bone morphology and function. This is mostly accomplished through bone remodeling, which involves the absorption of existing bone and the production of new bone, as conducted by osteoclasts and osteoblasts, respectively (Fig. 1). The balance between osteoblast and osteoclast activity maintains normal bone homeostasis [41]. The process of bone remodeling is mediated by the κ-β nuclear receptor (RANK)/RANK ligand (RANKL)/osteoprotegerin (OPG) axis. RANKL released by cells in the osteoblast lineage binds to its receptor RANK on precursor cells of osteoclasts. Then, RANK-RANKL binding drives precursor differentiation of precursor cells into osteoclasts, which merge to produce mature multinucleated osteoclasts.

Furthermore, osteoblasts release OPG, which functions as a decoy receptor for RANKL and inhibits RANK-RANKL interaction, reducing osteoclastogenesis [42]. Psychological stress activates the SNS. NE produced by nerve fibers activates β-ARs on osteoblasts, causing them to create interleukin 6 (IL-6) and interleukin 11 (IL-11), inhibiting bone formation. It also induces osteoblast lineage cells to produce RANKL, promoting osteoclast differentiation [43–45]. Neuropeptide Y (NPY), frequently generated with NE, negatively regulates bone development and contributes to bone loss [46, 47].

In addition to directly regulating bone homeostasis through neurotransmitter release, the SNS also exerts indirect control on bone via paracrine mechanisms involving EVs and cytokines. Sui et al. demonstrated that the application of isoproterenol (ISO), a beta-adrenergic receptor agonist, induced osteoblasts to transport EVs containing miR-21 to osteoclasts [48]. Subsequently, miR-21 played a crucial role in modulating osteoclastogenesis *in vivo* by inhibiting programmed cell death 4 (Pdcd4), resulting in bone loss, not through a direct reduction in osteoblast activity [49]. Elefteriou et al. revealed that sympathetic activation induced by leptin triggers bone resorption by upregulating the expression of the osteoclast differentiation factor RANKL in osteoblast progenitor cells [50]. This process is mediated through phosphorylated activating transcription factor 4 (ATF4), which plays an essential role in osteoblast differentiation and function, rather than via direct regulation of osteoclasts. Considering the indirect regulatory role of the SNS in bone homeostasis, various therapeutic strategies have been proposed. For instance, targeted delivery approaches specifically designed to silence osteoblastic miR-21 or employing (D-Asp8)-lipid nanoparticle-mediated targeted inhibition of osteoclastic miR-21, as well as clinically relevant drugs, show promise in restoring the balance of bone remodeling and alleviating ISO- and CVS-induced osteoporosis [48, 49]. Additionally, considering the significance of ADRB2, an effective, bone-specific pharmacological blockade targeting ADRB2 signaling would be highly beneficial in mitigating bone loss induced by sympathetic activation.

### Models of psychological stress and osteoporosis

Since the groundbreaking research by Holmes and Rahe in 1967, exploring the impact of life events on health, there has been a focused effort to establish precise animal models dedicated to studying the effects of psychological stress [51]. Currently, animal research models related to psychological stress are categorized into acute stress models, chronic stress models, and drug-induced models. Acute stress models, such as acute restraint stress and single prolonged stress, are designed to simulate situations in humans where experiences trigger intense negative emotions and a sense of trauma-inducing tension. These symptoms usually develop rapidly within minutes or hours–a quick reaction [52, 53]. Conversely, chronic stress models like social defeat, chronic variable stress, and trauma witness aim to simulate chronic changes in cognitive, emotional, physical, and behavioral aspects caused by a consistent sense of feeling pressured and overwhelmed over an extended period [52, 53].

Additionally, psychological stress models can also be established by administering drugs such as ISO and corticosterone (CORT) to mimic changes in hormones during psychological stress. The widespread use of this method is attributed to its singular treatment factor and predictable model establishment outcomes. Specific establishing methods of psychological stress related models have been summarized in Table 1. Moreover, the success of model establishment can be assessed through detections such as serum biomarker analysis, sucrose preference test, feeding experiments, social interaction trials, open field tests, forced swim tests, etc [48, 49, 79–83]. Among these psychological stress models, several have been confirmed to induce osteoporosis. For instance, in the study by Sui et al., models inducing psychological stress-related bone loss were established using chronic variable stress and ISO induction methods [48, 49]. Similarly, Zhang et al. detected reduced bone mass in the psychological stress model established through chronic social defeat stress methods [67].

**Table 1.** Rodent models of psychological stress

Models	Methods	Detections of sympathetic activation	Bone loss	Ref.
Time-dependent sensitization (TDS)	Exposure to brief, intense stimuli followed by subsequent pharmacological and non-pharmacological stressors.	Not enumerated	Not investigated	[54]
Electric shock (ES)	Inescapable electric foot shocks (shock duration and current depend on frequency), sometimes combined with situational reminders.	Open-field test, noise test, elevated plus-maze test	Not investigated	[55–57]
Underwater trauma (UT)	One-minute forced swim followed by 20–45 seconds of forced submersion in a water tank.	Transfer test	Not investigated	[58, 59].
Restraint stress (RS)	Immobilization on wooden boards or in a restraint container (e.g. syringe, wire mesh tube, 50 mL centrifuge tube) for 1–2 hours.	Elevated plus-maze test, biomarker assays	Yes	[60–63]
Single prolonged stress (SPS)	Two-hour restraint followed by a 20-minute forced swim and diethyl ether anesthesia until loss of consciousness.	Biomarker assays	Not investigated	[64]
Predator-based psychosocial stress (PPS)	Exposure to a natural predator or predator-related stimuli, sometimes coupled with unstable housing conditions for 3–4 weeks.	Elevated plus-maze test, noise test, radial-arm water maze, novel object recognition, biomarker assays	Not investigated	[65, 66]
Social defeat (SD)	Conspecific trained aggressor exposure for 6 hours daily for 5 or 10 days.	Biomarker assays, open-field test, forced swim test, sucrose preference test, tail suspension test, partition test ethogram evaluation, urine marking test	Yes	[67, 68]
Social isolation (SI)	Social isolation (complete or near-complete lack of social interaction) during 3–4 weeks.	Training test, contextual test, cued test, extinction test	Yes	[69, 70]
Trauma witness (TW)	Witnessing the social defeat event three times in one week.	Open-field test, elevated plus-maze test, light-dark exploration, forced swim stress, sucrose preference test, memory function test	Not investigated	[71]
Early life stress (ELS)	Separating animal pups from mothers for several hours daily during postnatal days 1–10, followed by re-exposure to stressors (e.g. underwater trauma) in adulthood.	Biomarker assays, elevated plus-maze test, acoustic startle response test, ultrasonic vocalizations, open-field test	Yes	[72–74]
Tail suspension (TS)	Suspension by the tail for 2–4 weeks.	Not enumerated	Yes	[75, 76]
Chronic variable stress (CVS)	Exposure to variable stressors for 5–9 weeks, e.g. overnight illumination, changing cage mates, food deprivation and so on.	Biomarker assays, sucrose preference test	Yes	[48, 49]
Chronic subordinate colony housing paradigm (CSC)	Housing in prolonged subordination to a dominant resident mouse for 19 consecutive days.	Biomarker assays, elevated plus-maze test	Not investigated	[77]
Drug induction	Daily intraperitoneal injections of ISO solution (10 mg/kg) for 4 weeks.	Biomarker assays	Yes	[48, 49]
	Daily subcutaneous injections of CORT solution (20 mg/kg) for 6 weeks.	Tail suspension test, forced swimming test, open field test	Not investigated	[78]

Currently, osteoporosis is categorized into primary and secondary types. Primary osteoporosis involves gonadectomy and aging, while secondary osteoporosis includes conditions such as diabetes, drug induction, disuse, psychological stress, and radiation exposure [84]. Table 2 outlines specific methodologies used to create models related to osteoporosis. Notably, some of these methods have been observed to trigger sympathetic activation (Table 2). For instance, Sui et al. uncovered that various osteoporotic mouse models such as ovariectomized mice and aging mice exhibited a significant increase in serum levels of the sympathetic activation marker–NE, but the mechanism remains unclear [48]. Moreover, the intricate distribution of nerves, blood vessels, and other components in bone, along with their interactions, remains a crucial area for investigation. In appropriate animal models, further elucidating whether and how the SNS regulates bone homeostasis through influencing blood vessels and other structures is of paramount importance in better understanding the relationship between psychological stress and osteoporosis.

**Table 2.** Rodent models of osteoporosis

Models	Methods	Sympathetic activation	Ref.
Gonadectomy	Ovariectomy (OVX) or orchidectomy (ORX).	Yes	[48]
Aging	Natural aging or using senescence accelerated-prone mice.		
Drug induction	Daily intraperitoneal injection of dexamethasone (20 mg/kg) for 4 weeks.		
Disuse	Tail suspension or hindlimb immobilization for 2 weeks.		
Psychological stress	Exposure to various stressors or daily ISO induction (10 mg/kg) for 4 weeks.		
Type 1 Diabetes	Daily intraperitoneal injection of streptozotocin (50 mg/kg) for 5 days.		
Type 2 Diabetes	Induction of insulin resistance via high-fat diet or other means.	Yes	[85]
Radiation	Focal radiation as a single dose of 20 Gy.	Not investigated	[86]

## Vascular regulation and bone homeostasis

### Vasculature coupled with bone formation and regeneration

Blood vessels are widely distributed in tissues and organs and perform the physiological function of blood transport and substance exchange by joining arteries, capillaries, and veins in sequence as a continuous and relatively closed tube system. Blood vessels also have a role in the physiological and pathological processes of bone, such as skeletal development, regeneration, hematopoiesis, and homeostasis regulation [87]. The connection between vascularization and osteogenesis is illustrated by endochondral ossification. Hypertrophic chondrocytes (HCs) begin hypoxia-inducible factor (HIF) signaling, enhance the expression of vascular endothelial growth factor (VEGF) target genes, and promote blood vessel formation [88] in the primary ossification center (POC). Matrix metalloproteinases (MMPs) are secreted by osteoclasts and endothelial cells (ECs), which degrade the extracellular matrix (ECM) and increase VEGF signaling. VEGF-mediated signaling promotes migration and proliferation of these cell types [89, 90] when ECs and osteoblasts express VEGFR2.

Researchers are increasingly interested in the relationship between angiogenesis and osteogenesis. Kusumbe et al. discovered in 2014 that different vascular types exhibit heterogeneity of ECs in bone. ECs in the metaphysis and endosteum had high expressions of CD31 and endomucin (EMCN) (CD31^hi^EMCN^hi^), identifying them as type H vessels. At the same time, ECs in the bone shaft had low CD31 and EMCN (CD31^lo^EMCN^lo^) expressions, identifying them as type L vessels [91]. Type H vessels are in tight contact with osteoprogenitor cells to provide necessary nutrients for bone formation, satisfy metabolic demands of bone development, and maintain bone homeostasis [92–94]. The expression levels of platelet-derived growth factor (PDGF), transforming growth factor (TGF), and other cytokines related to osteoprogenitor cell survival and proliferation were significantly higher in type H endothelium than in type L endothelium. Furthermore, in the metaphysis and endosteum, MSCs positive for platelet-derived growth factor receptor β (PDGFRβ), osteoblast-lineage cells positive for runt-related transcription factor 2 (RUNX2), and osteoblast-specific transcription factor (OSX) aggregate with type H endothelium. As a result, they exhibit a substantial spatial correlation, demonstrating the maintenance effect of type H endothelium on osteoblast-lineage cells.

### Vascular regulation by sympathetic nervous system

**Figure 2.**
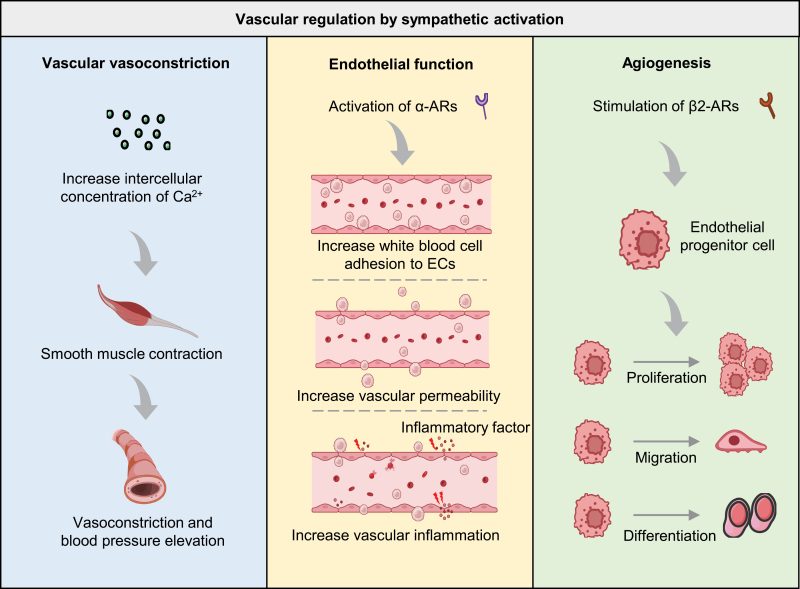
**Sympathetic activation governs various aspects of vascular function.**Sympathetic activation influences vascular vasoconstriction via Ca^2+^ concentration modulation. Endothelial functions, including adhesion, permeability, and inflammation, are regulated by the activation of α-ARs during sympathetic activation. Additionally, angiogenesis of endothelial progenitor cells (EPCs) is governed by β2-AR stimulation, which promotes EPC proliferation, migration, and differentiation. α-AR, alpha-adrenergic receptor; β2-AR, beta 2-adrenergic receptor; EPC, endothelial progenitor cell.

The regulation of angiotasis requires the intricate integration of several signals that promote vasodilation and vasoconstriction to maintain blood pressure and volume, ensuring appropriate oxygen supply to metabolically active tissues. There is a state of tensional balance between the vasoactive substances released by the endothelium and the neurotransmitters produced by sympathetic nerve terminals [95]. The endothelium produces and releases numerous vasoactive substances in normal conditions, including endothelin 1 (ET-1) and angiotensin II (Ang II) for vasoconstriction and nitric oxide (NO), prostacyclin (PGI2), and endothelium-derived hyperpolarizing factor (EDHF) for vasodilation [96]. Under sympathoexcitation, the released NE causes systemic vasoconstriction and increases blood pressure by causing smooth muscle contraction via increasing the intercellular concentration of Ca^2+^ (Fig. 2). The endothelium inhibits sympathetic activity by promoting the metabolism and breakdown of NE to relieve vasoconstriction, providing a physical barrier to prevent NE diffusion into the bloodstream [97]. Moreover, in the coronary and large arteries, the activation of α2-ARs and β-ARs expressed on ECs leads to NO release and further vasodilation [98].

The SNS has been found to not only affect vasoconstriction and vasodilation, but also influence endothelial function and angiogenesis through Ars, with the continuous deepening of research on neurovascular interactions (Fig. 2). Angiogenesis refers to the biological process of producing new blood vessels on existing vasculature, often by sprouting and intussusception, and it is critical in physiological and pathological processes [99, 100]. Intussusceptive angiogenesis is the process by which a single blood vessel divides to form two lumens, also known as splitting angiogenesis [101, 102]. Sprouting angiogenesis involves forming new blood vessels from existing ones by endothelial sprouting [103, 104]. Studies have shown that the activation of α-ARs leads to endothelial dysfunction characterized by increased white blood cell adhesion to ECs, vascular inflammation, and increased vascular permeability [105–107]. Furthermore, stimulating β2-ARs on endothelial progenitor cells (EPCs) leads to its proliferation, migration, differentiation, and angiogenesis [108].

### Specialized neurovascular coupling regulated by sympathetic nervous system

Bone blood vessels are densely distributed and continually transport oxygen, nutrients, and waste. At the same time, they also play a critical role in physiological and pathological processes. The discovery of type H vessels confirms the heterogeneity of ECs and provides evidence supporting the theory of angiogenesis–osteogenesis coupling. Research on promoting bone formation by type H vessels is increasing, highlighting the importance of specialized vasculature in bone homeostasis and regeneration.

**Figure 3.**
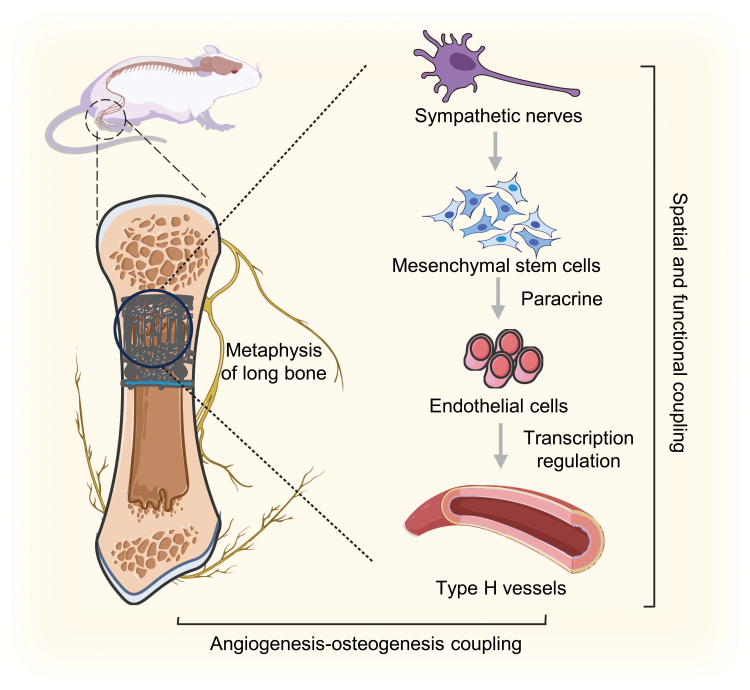
**Neurovascular coupling and their regulation in bone homeostasis and regeneration under sympathetic activation.**Close spatial connections and functional coupling exist between sympathetic nerves and type H vessels in the metaphysis. Sympathetic activation stimulates MSCs to transcriptionally regulate type H vessel angiogenesis via paracrine effects, subsequently impacting bone homeostasis and regeneration through the coupling of angiogenesis and osteogenesis.

Notably, close spatial connections between sympathetic nerves and type H vessels in metaphysis have been proven, in which ADRBs, particularly ADRB2, negatively regulate type H vasculature and bone homeostasis (Fig. 3).

Sympathoexcitation induces a reduction in type H vessels and bone mass *in vivo,* primarily through indirect sympathetic modulation of angiogenesis mediated by the paracrine effects of mesenchymal stem cells (MSCs). This regulatory mechanism involves alterations in the transcriptional activity of several angiogenic genes within ECs.

Xu et al. elucidated a distinct type of neurovascular coupling, proposing a novel approach centered on neural signaling and targeted vasculature for osteoanabolic therapies. Although the mechanisms by which pericytes respond to sympathetic activation and influence the formation of type H vessels through paracrine mechanisms remain unclear, this indirect regulatory mechanism may provide a new perspective for understanding neurovascular coupling in bone. EVs, as a crucial mode of intercellular signaling, have the capability to indirectly respond to sympathetic activation, thereby regulating bone homeostasis. Hence, investigating whether and how they participate in the sympathetic regulation of vasculature holds significant importance in further elucidating the mechanisms behind sympathoexcitation in regulating specialized type H endothelium and its potential effects on bone homeostasis and regeneration under psychological stress.

## Extracellular vesicles and vascular regulation

### Functions and applications of extracellular vesicles

EVs are small membrane-bound vesicles secreted actively by cells [109]. Initially, they were considered a form of waste excreted by cells. However, Harding and Pan discovered in 1983 that reticulocytes secreted small vesicles during maturation, transferring transferrin to the extracellular space [110, 111]. Later, Raposo et al. found that EVs secreted by B lymphocytes activated T lymphocytes and participated in immune regulation [112]. Since then, the functions of EVs have gained more attention. EVs are categorized according to their size and are distinguished as exosomes (40–160 nm), microvesicles (100–1000 nm), and apoptotic bodies (1000–5000 nm) [113, 114]. EVs remove excess or unnecessary components from cells to maintain cellular homeostasis and play a vital role in regulating intercellular communication by carrying proteins, nucleic acids, small molecules, and structural signaling molecules [115–117]. The key and complex processes that mediate intercellular communication include EVs recognition, binding, cellular uptake, and intracellular transport. EVs possess integrins, ECM proteins, lectins, proteoglycans, and glycolipids, enabling them to recognize and bind to specific receptors on target cells [118]. Subsequently, EVs mainly deliver their contents to the interior of cells by binding to receptors on target cells, fusing with the target cell plasma membranes, or being internalized, exerting biological effects.

The potential of EVs for diagnosing and treating several diseases, including neurodegenerative diseases and cardiovascular diseases, has recently been revealed [119–122]. EVs are abundant in blood and other bodily fluids, and their amounts and cargo of nucleic acids, proteins, lipids, and other substances reflect the physiological and pathological processes of the body, prompting them to be examined as biomarkers for various diseases [123]. Regarding treatment, EVs include functional substances, such as exogenous drugs, therapeutic RNA molecules, or proteins. They have been changed on their surface to create biological effects on receptor cells, allowing them to be used as a functional therapy for specific diseases [124–126].

### Angiogenesis regulation by EVs

EVs control several physiological and pathological processes by delivering bioactive molecules. Numerous studies have shown that EVs play a critical role in angiogenesis regulation. For example, EVs produced from ECs contain β1 integrin, MMP-2, and MMP-9, which promote EC invasion and capillary-like structure formation [127]. EVs derived from endothelial colony-forming cells (ECFCs), which possess clear endothelial differentiation potential and proliferation activity, engage with α4 and β1 integrins to activate angiogenesis by conveying mRNA from the eNOS and PI3K/Akt signaling pathways [128, 129]. Recently, EVs have been considered potential mediators of remote ischemic adaptation and general protective signals. The ability of EVs to regulate angiogenesis offers new therapeutic opportunities for regenerative medicine and tissue engineering [130, 131]. Intramyocardial treatment with EVs can increase the perfusion of ischemic myocardial tissue by inducing arteriole and capillary growth in chronic ischemic heart disease [132]. EVs derived from ECFCs prevented capillary rarefaction and subsequent tissue damage in a rat model of renal ischemia-reperfusion injury [133]. EVs secreted by ECFCs enhance angiogenesis and accelerate wound healing by strengthening blood vessels at the wound site [134].

Furthermore, MSC-EVs has been confirmed to promote the formation of type H vessels in bone. Zhuang et al. induced the proangiogenic activity of MSCs through a hypoxic microenvironment. They confirmed that miR-210-3p, upregulated in small EVs derived from hypoxic MSCs, downregulated EFNA3 expression and enhanced the phosphorylation of the PI3K/AKT pathway, which facilitated the proliferation, migration, and angiogenesis abilities of ECs and ultimately enhanced bone regeneration and type H vessel formation in the critical size calvarial bone defect model [135]. Not only in bone, MSC-EVs have also been proven to promote the formation of type H vessels in the skin. Liu et al. demonstrated that EVs derived from engineered mesenchymal stem/stromal cell aggregates enriched angiogenic proteins. These proteins activated the Notch-related signaling pathway, promoting the formation of type H vessels in diabetic wounds, and ultimately facilitating their healing [136].

In summary, EVs are intercellular communication mediators used for diagnosing and treating various diseases. EVs produced from blood-related cells or other sources have been demonstrated to influence angiogenesis and have effective therapeutic benefits. However, it is still unknown whether sympathetic under psychological stress affects the role of EVs in angiogenesis regulation. Type H vessels are tightly coupled with bone homeostasis and regeneration, and it is unclear how they respond to the regulation of endogenous EVs and the effects of exogenous EVs on type H vessel maintenance in the context of sympathetic activation. Therefore, it is critical to investigate the role of EVs in specialized regeneration-related vascular structure regulation under sympathetic activation to develop therapy methods for bone loss caused by psychological stress.

## MSC-EVs regulate angiogenesis and bone regeneration

### MSC-EVs in angiogenesis regulation

**Figure 4.**
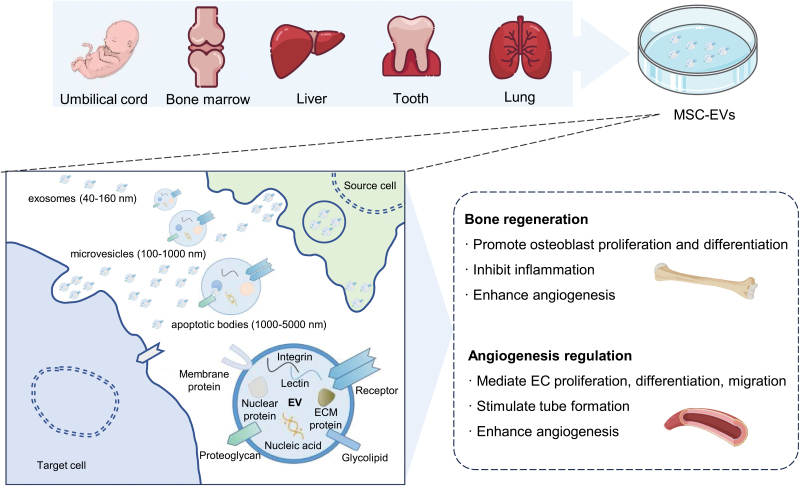
**Regulation of angiogenesis and bone regeneration by MSC-EVs.**MSCs are derived from various tissues, including the umbilical cord, bone marrow, liver, tooth, and lung. MSC-EVs encompass exosomes, microvesicles, and apoptotic bodies, containing diverse components like proteins and nucleic acids. MSC-EVs are employed for promoting bone regeneration and regulating angiogenesis. MSC, mesenchymal stem cells; MSC-EVs, mesenchymal stem cell extracellular vesicles.

MSCs are a type of adult non-hematopoietic stem cell discovered by Friedenstein et al. in bone marrow. They possess characteristics such as adhesion to plastic, self-renewal, clonogenicity, and multipotential differentiation abilities [137, 138]. MSCs are isolated from various body parts, including blood, bone marrow, liver, umbilical cord, periodontal ligament, lung, and adipose tissue [139] (Fig. 4). During tissue regeneration, it was initially believed that MSCs exerted therapeutic effects by migrating to the injury site, engrafting, and subsequently differentiating into the cells required for tissue regeneration. However, increasing studies have shown that the therapeutic benefits of MSCs are attributed to their differentiation and the active substances they secrete, especially the role of EVs, which has received extensive attention [140–142]. MSC-EVs transmit information to damaged cells or tissues, participate in tissue regeneration and functional repair, regulate extracellular matrix remodeling, limit local inflammation, and regulate immune responses [143–145]. Numerous studies have found that EVs released by MSCs have therapeutic potential in repairing diseases and functional damage in various tissues and organs, such as the lung, kidney, liver, nervous system, bone, cartilage, and heart [146–152].

Several studies have demonstrated the significant role of MSC-EVs in promoting angiogenesis [153, 154] (Fig. 4). For instance, under hypoxic stimulation, bone marrow-derived MSC-EVs are absorbed by ECs, thereby promoting angiogenesis and enhancing cardiac function in rat myocardial infarction models [155]. After low oxygen treatment, umbilical cord-derived MSC-EVs improve the formation of capillary-like structures and promote blood flow recovery in rat models of hindlimb ischemia [156, 157]. Moreover, umbilical cord-derived MSC-EVs overexpressing Akt stimulate EC migration, proliferation, and tube formation *in vitro*, increasing blood vessel formation *in vivo* [158]. Similarly, stem cells from human exfoliated deciduous teeth (SHED)-derived MSC-EVs mediate endothelial cell differentiation and enhance angiogenesis by transferring miR-26a via the TGF-β/SMAD2/3 signaling pathway [159].

### MSC-EVs in bone regeneration

In recent years, multiple studies have confirmed that MSC-EVs directly promote bone cell proliferation, differentiation, and angiogenesis while inhibiting inflammatory responses in various pathological conditions [160–163] (Fig. 4). Treatment in the osteonecrosis model with MSC–EV increased trabecular bone cell numbers, decreased apoptotic cell numbers, and reduced adipose tissue [164]. MSC-EVs in the fracture model promoted bone callus formation and enhanced bone healing [165]. Profound remodeling of newly formed bone in the osteoporosis model was observed with MSC-EVs treatment. In contrast, the control group without MSC-EVs treatment exhibited bone loss or minimal mineralization and bone formation [166]. Furthermore, MSC-EVs promote angiogenesis in osteoporotic rats, which is beneficial for bone formation [163]. More experiments have also revealed the potential mechanism of MSC-EVs in bone regeneration therapy. MSC-EVs may promote the osteogenic differentiation of bone marrow MSCs and induce osteogenesis by regulating the PI3K/Akt signaling pathway [167]. Additionally, MSC-EVs stimulate the proliferation and differentiation of osteoblasts by regulating the BMP/Smad/Wnt signaling pathway, thereby promoting osteogenesis [165, 166].

Although MSC-EVs have shown great potential in regenerative therapy, their application is limited by low natural production. In recent years, engineering techniques have been developed to modify MSC-EVs to avoid potential pathogenic risks and enhance their efficacy and production to meet the demand for MSC-EVs in diseases [167]. Engineering MSC-EVs involves surface modification and content loading. Surface modification is achieved through cosmetic cell modification and non-covalent or covalent membrane binding to make MSC-EVs target specific cells or reduce the probability of being cleared [168]. Loading functional components, including nucleic acids, proteins, and small molecules, into the lipid membrane bilayer structure on the surface of MSC-EVs and the hydrophilic core surrounded by the lipid membrane is known as content loading. Passive loading involves co-incubating functional therapeutic substances with EVs or donor cells, whereas active loading involves ultrasonic treatment, extrusion procedures, electroporation, multiple freeze-thaw, and other techniques [152, 169, 170]. While passive loading is straightforward and maintains the EV membranes intact, it has poor loading efficiency. On the other hand, active loading can provide a high loading efficiency for hydrophilic or macromolecular compounds but might result in issues, such as damage to membrane integrity or condensation [171–173].

Overall, MSC-EVs derived from MSCs with multipotent differentiation potential promote tissue regeneration, limit local inflammation, regulate immune responses, and effectively promote angiogenesis and bone regeneration. However, it is unclear how MSC-EVs respond to and exert their regenerative-promoting effects in the overall environment under sympathetic activation. The low natural production and lack of specificity of MSC-EVs limit their broad application in regenerative medicine. Therefore, engineering modifications of MSC-EVs to improve their targeting and therapeutic effects will better meet the demand for MSC-EVs in bone regeneration. Modified MSC-EVs with proangiogenic and pro-osteogenic effects will provide new options and directions for treating bone loss under psychological stress.

## Summary and prospectives

Psychological stress is closely related to the development and progression of several diseases, including bone loss and osteoporosis. SNS, one of the neuroendocrine foundations of psychological stress, has been shown to significantly contribute to this process via overactivation or malfunction. However, the mechanism underlying sympathetic activation-induced bone loss remains unknown, and no viable therapy strategies are available. Recent research suggests sympathetic activation modulates bone and vascular cell homeostasis by acting on bone cells and ECs. Notably, the close distribution and mutual interaction of nerves and blood vessels in bone suggest that sympathetic activation might participate in bone regeneration by influencing vascular function. Identifying type H vessels, a specialized vasculature linked to bone regeneration highlights the significance of vascularization in modulating the response to sympathetic activation-induced bone loss. An emerging therapy based on EVs, namely MSC–EVs, is proposed to repair bone loss under psychological stress, with favorable proangiogenic and pro-osteogenic effects. We also propose engineering modifications to improve the targeting and therapeutic benefits of MSC-EVs to develop an effective novel technique for treating osteoporosis under psychological stress.

## References

[1] Baum A. Stress, intrusive imagery, and chronic distress. Health Psychol 1990;9:653–75. 2286178 10.1037//0278-6133.9.6.653

[2] Willmore L, Cameron C, Yang J, et al. Behavioural and dopaminergic signatures of resilience. Nature 2022;611:124–32. 36261520 10.1038/s41586-022-05328-2 PMC10026178

[3] Woolston C. Stress and uncertainty drag down graduate students’ satisfaction. Nature 2022;610:805–8. 36280727 10.1038/d41586-022-03394-0

[4] Haslam C, Haslam SA, Jetten J, et al. Life change, social identity, and health. Annu Rev Psychol 2021;72:635–61. 32886584 10.1146/annurev-psych-060120-111721

[5] Cohen S, Janicki-Deverts D, Miller GE. Psychological stress and disease. JAMA 2007;298:1685–7. 17925521 10.1001/jama.298.14.1685

[6] Baron KG, Reid KJ. Circadian misalignment and health. Int Rev Psychiatry 2014;26:139–54. 24892891 10.3109/09540261.2014.911149 PMC4677771

[7] Biddie SC, Conway-Campbell BL, Lightman SL. Dynamic regulation of glucocorticoid signalling in health and disease. Rheumatology 2012;51:403–12. 21891790 10.1093/rheumatology/ker215 PMC3281495

[8] Chrousos GP. Stress and disorders of the stress system. Nat Rev Endocrinol 2009;5:374–81. 19488073 10.1038/nrendo.2009.106

[9] Cruz-Pereira JS, Rea K, Nolan YM, et al. Depression’s unholy trinity: dysregulated stress, immunity, and the microbiome. Annu Rev Psychol 2020;71:49–78. 31567042 10.1146/annurev-psych-122216-011613

[10] De Kloet ER, Vreugdenhil E, Oitzl MS, et al. Brain corticosteroid receptor balance in health and disease. Endocr Rev 1998;19:269–301. 9626555 10.1210/edrv.19.3.0331

[11] Miller GE, Cohen S, Ritchey AK. Chronic psychological stress and the regulation of pro-inflammatory cytokines: a glucocorticoid-resistance model. Health Psychol 2002;21:531–41. 12433005 10.1037//0278-6133.21.6.531

[12] Potter GD, Skene DJ, Arendt J, et al. Circadian rhythm and sleep disruption: Causes, metabolic consequences, and countermeasures. Endocr Rev 2016;37:584–608. 27763782 10.1210/er.2016-1083 PMC5142605

[13] Compston JE, McClung MR, Leslie WD. Osteoporosis. Lancet 2019;393:364–76. 30696576 10.1016/S0140-6736(18)32112-3

[14] Sarafrazi N, Wambogo EA, Shepherd JA. Osteoporosis or low bone mass in older adults: United States, 2017-2018. NCHS Data Brief 2021;405:1–8. 34029181

[15] Ng JS, Chin KY. Potential mechanisms linking psychological stress to bone health. Int J Med Sci 2021;18:604–14. 33437195 10.7150/ijms.50680 PMC7797546

[16] Bab I, Yirmiya R. Depression, selective serotonin reuptake inhibitors, and osteoporosis. Curr Osteoporos Rep 2010;8:185–91. 20809204 10.1007/s11914-010-0026-z

[17] Erez HB, Weller A, Vaisman N, et al. The relationship of depression, anxiety and stress with low bone mineral density in post-menopausal women. Arch Osteoporos 2012;7:247–55. 23095987 10.1007/s11657-012-0105-0

[18] Cizza G, Primma S, Csako G. Depression as a risk factor for osteoporosis. Trends Endocrinol Metab 2009;20:367–73. 19747841 10.1016/j.tem.2009.05.003 PMC2764354

[19] Pedersen AB, Baggesen LM, Ehrenstein V, et al. Perceived stress and risk of any osteoporotic fracture. Osteoporos Int 2016;27:2035–45. 26786258 10.1007/s00198-016-3490-1

[20] El-Gabalawy R, Blaney C, Tsai J, et al. Physical health conditions associated with full and subthreshold PTSD in U.S. military veterans: Results from the National Health and Resilience in Veterans Study. J Affect Disord 2018;227:849–53. 29689700 10.1016/j.jad.2017.11.058 PMC6269149

[21] Rauma PH, Honkanen RJ, Williams LJ, et al. Effects of antidepressants on postmenopausal bone loss – A 5-year longitudinal study from the OSTPRE cohort. Bone 2016;89:25–31. 27179631 10.1016/j.bone.2016.05.003

[22] Russell G, Lightman S. The human stress response. Nat Rev Endocrinol 2019;15:525–34. 31249398 10.1038/s41574-019-0228-0

[23] O’Connor DB, Thayer JF, Vedhara K. Stress and health: a review of psychobiological processes. Annu Rev Psychol 2021;72:663–88. 32886587 10.1146/annurev-psych-062520-122331

[24] al’Absi M, Wittmers LE, Jr. Enhanced adrenocortical responses to stress in hypertension-prone men and women. Ann Behav Med 2003;25:25–33. 12581933 10.1207/S15324796ABM2501_04

[25] Adam EK, Quinn ME, Tavernier R, et al. Diurnal cortisol slopes and mental and physical health outcomes: a systematic review and meta-analysis. Psychoneuroendocrinology 2017;83:25–41. 28578301 10.1016/j.psyneuen.2017.05.018 PMC5568897

[26] McEwen BS. Protective and damaging effects of stress mediators. N Engl J Med 1998;338:171–9. 9428819 10.1056/NEJM199801153380307

[27] Espinosa-Medina I, Saha O, Boismoreau F, et al. The sacral autonomic outflow is sympathetic. Science 2016;354:893–7. 27856909 10.1126/science.aah5454 PMC6326350

[28] Elefteriou F. Impact of the autonomic nervous system on the skeleton. Physiol Rev 2018;98:1083–112. 29717928 10.1152/physrev.00014.2017 PMC6088147

[29] Tourtellotte WG. Axon transport and neuropathy: relevant perspectives on the etiopathogenesis of familial dysautonomia. Am J Pathol 2016;186:489–99. 26724390 10.1016/j.ajpath.2015.10.022 PMC4816694

[30] Hasan W. Autonomic cardiac innervation: development and adult plasticity. Organogenesis 2013;9:176–93. 23872607 10.4161/org.24892 PMC3896589

[31] Goldstein DS, Robertson D, Esler M, et al. Dysautonomias: clinical disorders of the autonomic nervous system. Ann Intern Med 2002;137:753–63. 12416949 10.7326/0003-4819-137-9-200211050-00011

[32] Saravia F, Homo-Delarche F. Is innervation an early target in autoimmune diabetes*?* Trends Immunol 2003;24:574–9. 14596878 10.1016/j.it.2003.09.010

[33] Hanoun M, Maryanovich M, Arnal-Estape A, et al. Neural regulation of hematopoiesis, inflammation, and cancer. Neuron 2015;86:360–73. 25905810 10.1016/j.neuron.2015.01.026 PMC4416657

[34] Karsenty G, Olson EN. Bone and muscle endocrine functions: unexpected paradigms of inter-organ communication. Cell 2016;164:1248–56. 26967290 10.1016/j.cell.2016.02.043 PMC4797632

[35] Cappariello A, Ponzetti M, Rucci N. The “soft” side of the bone: unveiling its endocrine functions. Horm Mol Biol Clin Investig 2016;28:5–20. 10.1515/hmbci-2016-0009 27107839

[36] Ducy P, Amling M, Takeda S, et al. Leptin inhibits bone formation through a hypothalamic relay: a central control of bone mass. Cell 2000;100:197–207. 10660043 10.1016/s0092-8674(00)81558-5

[37] Sato S, Hanada R, Kimura A, et al. Central control of bone remodeling by neuromedin *U*. Nat Med 2007;13:1234–40. 17873881 10.1038/nm1640

[38] Togari A. Adrenergic regulation of bone metabolism: possible involvement of sympathetic innervation of osteoblastic and osteoclastic cells. Microsc Res Tech 2002;58:77–84. 12203706 10.1002/jemt.10121

[39] Asmus SE, Parsons S, Landis SC. Developmental changes in the transmitter properties of sympathetic neurons that innervate the periosteum. J Neurosci 2000;20:1495–504. 10662839 10.1523/JNEUROSCI.20-04-01495.2000 PMC6772371

[40] Nagata A, Tanaka T, Minezawa A, et al. cAMP activation by PACAP/VIP stimulates IL-6 release and inhibits osteoblastic differentiation through VPAC2 receptor in osteoblastic MC3T3 cells. J Cell Physiol 2009;221:75–83. 19496170 10.1002/jcp.21831

[41] Sims NA, Martin TJ. Coupling signals between the osteoclast and osteoblast: How are messages transmitted between these temporary visitors to the bone surface*?* Front Endocrinol 2015;6:41. 10.3389/fendo.2015.00041 PMC4371744 25852649

[42] Rachner TD, Khosla S, Hofbauer LC. Osteoporosis: now and the future. Lancet 2011;377:1276–87. 21450337 10.1016/S0140-6736(10)62349-5 PMC3555696

[43] Yao Q, Liang H, Huang B, et al. Beta-adrenergic signaling affect osteoclastogenesis via osteocytic MLO-Y4 cells’ RANKL production. Biochem Biophys Res Commun 2017;488:634–40. 27823934 10.1016/j.bbrc.2016.11.011

[44] Ma Y, Nyman JS, Tao H, et al. beta2-Adrenergic receptor signaling in osteoblasts contributes to the catabolic effect of glucocorticoids on bone. Endocrinology 2011;152:1412–22. 21266510 10.1210/en.2010-0881 PMC3060633

[45] Kondo A, Mogi M, Koshihara Y, et al. Signal transduction system for interleukin-6 and interleukin-11 synthesis stimulated by epinephrine in human osteoblasts and human osteogenic sarcoma cells. Biochem Pharmacol 2001;61:319–26. 11172736 10.1016/s0006-2952(00)00544-x

[46] Yahara M, Tei K, Tamura M. Inhibition of neuropeptide Y Y1 receptor induces osteoblast differentiation in MC3T3-E1 cells. Mol Med Rep 2017;16:2779–84. 28656295 10.3892/mmr.2017.6866

[47] Wee NKY, Sinder BP, Novak S, et al. Skeletal phenotype of the neuropeptide Y knockout mouse. Neuropeptides 2019;73:78–88. 30522780 10.1016/j.npep.2018.11.009 PMC6326877

[48] Hu CH, Sui BD, Liu J, et al. Sympathetic neurostress drives osteoblastic exosomal MiR-21 transfer to disrupt bone homeostasis and promote osteopenia. Small Methods 2022;6:e2100763. 35312228 10.1002/smtd.202100763

[49] Sui B, Liu J, Zheng C, et al. Targeted inhibition of osteoclastogenesis reveals the pathogenesis and therapeutics of bone loss under sympathetic neurostress. Int J Oral Sci 2022;14:39. 35915088 10.1038/s41368-022-00193-1 PMC9343357

[50] Elefteriou F, Ahn JD, Takeda S, et al. Leptin regulation of bone resorption by the sympathetic nervous system and CART. Nature 2005;434:514–20. 15724149 10.1038/nature03398

[51] Holmes TH, Rahe RH. The social readjustment rating scale. J Psychosom Res 1967;11:213–8. 6059863 10.1016/0022-3999(67)90010-4

[52] McEwen BS. Physiology and neurobiology of stress and adaptation: central role of the brain. Physiol Rev 2007;87:873–904. 17615391 10.1152/physrev.00041.2006

[53] Wirtz PH, von Kanel R. Psychological stress, inflammation, and coronary heart disease. Curr Cardiol Rep 2017;19:111. 28932967 10.1007/s11886-017-0919-x

[54] Antelman SM, DeGiovanni LA, Kocan D. A single exposure to cocaine or immobilization stress provides extremely long-lasting, selective protection against sudden cardiac death from tetracaine. Life Sci 1989;44:201–7. 2915599 10.1016/0024-3205(89)90596-1

[55] Van Dijken HH, Van der Heyden JA, Mos J, et al. Inescapable footshocks induce progressive and long-lasting behavioural changes in male rats. Physiol Behav 1992;51:787–94. 1594677 10.1016/0031-9384(92)90117-k

[56] Pynoos RS, Ritzmann RF, Steinberg AM, et al. A behavioral animal model of posttraumatic stress disorder featuring repeated exposure to situational reminders. Biol Psychiatry 1996;39:129–34. 8717611 10.1016/0006-3223(95)00088-7

[57] Siegmund A, Wotjak CT. A mouse model of posttraumatic stress disorder that distinguishes between conditioned and sensitised fear. J Psychiatr Res 2007;41:848–60. 17027033 10.1016/j.jpsychires.2006.07.017

[58] Morris R. Developments of a water-maze procedure for studying spatial learning in the rat. J Neurosci Methods 1984;11:47–60. 6471907 10.1016/0165-0270(84)90007-4

[59] Richter-Levin G. Acute and long-term behavioral correlates of underwater trauma--potential relevance to stress and post-stress syndromes. Psychiatry Res 1998;79:73–83. 9676829 10.1016/s0165-1781(98)00030-4

[60] Balkaya M, Prinz V, Custodis F, et al. Stress worsens endothelial function and ischemic stroke via glucocorticoids. Stroke 2011;42:3258–64. 21921276 10.1161/STROKEAHA.110.607705

[61] Gameiro GH, Gameiro PH, Andrade Ada S, et al. Nociception- and anxiety-like behavior in rats submitted to different periods of restraint stress. Physiol Behav 2006;87:643–9. 16488452 10.1016/j.physbeh.2005.12.007

[62] Nakajima K, Hamada N, Takahashi Y, et al. Restraint stress enhances alveolar bone loss in an experimental rat model. J Periodontal Res 2006;41:527–34. 17076777 10.1111/j.1600-0765.2006.00901.x

[63] Takeda O, Toyama T, Watanabe K, et al. Ameliorating effects of Juzentaihoto on restraint stress and P. gingivalis-induced alveolar bone loss. Arch Oral Biol 2014;59:1130–8. 25064760 10.1016/j.archoralbio.2014.06.010

[64] Liberzon I, Krstov M, Young EA. Stress-restress: effects on ACTH and fast feedback. Psychoneuroendocrinology 1997;22:443–53. 9364622 10.1016/s0306-4530(97)00044-9

[65] Adamec RE, Shallow T. Lasting effects on rodent anxiety of a single exposure to a cat. Physiol Behav 1993;54:101–9. 8327588 10.1016/0031-9384(93)90050-p

[66] Zoladz PR, Conrad CD, Fleshner M, et al. Acute episodes of predator exposure in conjunction with chronic social instability as an animal model of post-traumatic stress disorder. Stress 2008;11:259–81. 18574787 10.1080/10253890701768613 PMC2535807

[67] Zhang J, Fujita Y, Chang L, et al. Beneficial effects of anti-RANKL antibody in depression-like phenotype, inflammatory bone markers, and bone mineral density in male susceptible mice after chronic social defeat stress. Behav Brain Res 2020;379:112397. 31790783 10.1016/j.bbr.2019.112397

[68] Hammamieh R, Chakraborty N, De Lima TC, et al. Murine model of repeated exposures to conspecific trained aggressors simulates features of post-traumatic stress disorder. Behav Brain Res 2012;235:55–66. 22824590 10.1016/j.bbr.2012.07.022

[69] Pibiri F, Nelson M, Guidotti A, et al. Decreased corticolimbic allopregnanolone expression during social isolation enhances contextual fear: a model relevant for posttraumatic stress disorder. Proc Natl Acad Sci U S A 2008;105:5567–72. 18391192 10.1073/pnas.0801853105 PMC2291140

[70] Mountain RV, Langlais AL, Hu D, et al. Social isolation through single housing negatively affects trabecular and cortical bone in adult male, but not female, C57BL/6J mice. Bone 2023;172:116762. 37044360 10.1016/j.bone.2023.116762 PMC10084633

[71] Patki G, Solanki N, Salim S. Witnessing traumatic events causes severe behavioral impairments in rats. Int J Neuropsychopharmacol 2014;17:2017–29. 24887568 10.1017/S1461145714000923 PMC4318493

[72] Ardi Z, Albrecht A, Richter-Levin A, et al. Behavioral profiling as a translational approach in an animal model of posttraumatic stress disorder. Neurobiol Dis 2016;88:139–47. 26804028 10.1016/j.nbd.2016.01.012

[73] Kalinichev M, Easterling KW, Plotsky PM, et al. Long-lasting changes in stress-induced corticosterone response and anxiety-like behaviors as a consequence of neonatal maternal separation in Long-Evans rats. Pharmacol Biochem Behav 2002;73:131–40. 12076732 10.1016/s0091-3057(02)00781-5

[74] Bertolini Botelho MC, Cintra LTA, da Silva CC, et al. Early life stress exacerbates bone resorption and inhibits anxiety-like behaviour induced by apical periodontitis in rats. Int Endod J 2023;56:203–12. 36310440 10.1111/iej.13857

[75] Steru L, Chermat R, Thierry B, et al. The tail suspension test: a new method for screening antidepressants in mice. Psychopharmacology 1985;85:367–70. 3923523 10.1007/BF00428203

[76] Hu Y, Zhang Y, Ni CY, et al. Human umbilical cord mesenchymal stromal cells-derived extracellular vesicles exert potent bone protective effects by CLEC11A-mediated regulation of bone metabolism. Theranostics 2020;10:2293–308. 32089743 10.7150/thno.39238 PMC7019162

[77] Reber SO, Birkeneder L, Veenema AH, et al. Adrenal insufficiency and colonic inflammation after a novel chronic psycho-social stress paradigm in mice: implications and mechanisms. Endocrinology 2007;148:670–82. 17110427 10.1210/en.2006-0983

[78] Xie X, Shen Q, Yu C, et al. Depression-like behaviors are accompanied by disrupted mitochondrial energy metabolism in chronic corticosterone-induced mice. J Steroid Biochem Mol Biol 2020;200:105607. 32045672 10.1016/j.jsbmb.2020.105607

[79] Fetcho RN, Hall BS, Estrin DJ, et al. Regulation of social interaction in mice by a frontostriatal circuit modulated by established hierarchical relationships. Nat Commun 2023;14:2487. 37120443 10.1038/s41467-023-37460-6 PMC10148889

[80] Francois M, Canal Delgado I, Shargorodsky N, et al. Assessing the effects of stress on feeding behaviors in laboratory mice. Elife 2022;11:e70271. 35167441 10.7554/eLife.70271 PMC8846584

[81] Lee EH, Park JY, Kwon HJ, et al. Repeated exposure with short-term behavioral stress resolves pre-existing stress-induced depressive-like behavior in mice. Nat Commun 2021;12:6682. 34795225 10.1038/s41467-021-26968-4 PMC8602389

[82] Liu MY, Yin CY, Zhu LJ, et al. Sucrose preference test for measurement of stress-induced anhedonia in mice. Nat Protoc 2018;13:1686–98. 29988104 10.1038/s41596-018-0011-z

[83] Shi X, Gao Y, Song L, et al. Sulfur dioxide derivatives produce antidepressant- and anxiolytic-like effects in mice. Neuropharmacology 2020;176:108252. 32712276 10.1016/j.neuropharm.2020.108252

[84] Sui B, Hu C, Liao L, et al. Mesenchymal progenitors in osteopenias of diverse pathologies: differential characteristics in the common shift from osteoblastogenesis to adipogenesis. Sci Rep 2016;6:30186. 27443833 10.1038/srep30186 PMC4957106

[85] Young BE, Holwerda SW, Vranish JR, et al. Sympathetic transduction in type 2 diabetes mellitus. Hypertension 2019;74:201–7. 31188673 10.1161/HYPERTENSIONAHA.119.12928 PMC6594391

[86] Rana T, Schultz MA, Freeman ML, et al. Loss of Nrf2 accelerates ionizing radiation-induced bone loss by upregulating RANKL. Free Radic Biol Med 2012;53:2298–307. 23085426 10.1016/j.freeradbiomed.2012.10.536 PMC3762920

[87] Filipowska J, Tomaszewski KA, Niedzwiedzki L, et al. The role of vasculature in bone development, regeneration and proper systemic functioning. Angiogenesis 2017;20:291–302. 28194536 10.1007/s10456-017-9541-1 PMC5511612

[88] Maes C, Carmeliet G, Schipani E. Hypoxia-driven pathways in bone development, regeneration and disease. Nat Rev Rheumatol 2012;8:358–66. 22450551 10.1038/nrrheum.2012.36

[89] Clarkin C, Olsen BR. On bone-forming cells and blood vessels in bone development. Cell Metab 2010;12:314–6. 20889123 10.1016/j.cmet.2010.09.009 PMC3001285

[90] Duan X, Murata Y, Liu Y, et al. Vegfa regulates perichondrial vascularity and osteoblast differentiation in bone development. Development 2015;142:1984–91. 25977369 10.1242/dev.117952 PMC4460736

[91] Kusumbe AP, Ramasamy SK, Adams RH. Coupling of angiogenesis and osteogenesis by a specific vessel subtype in bone. Nature 2014;507:323–8. 24646994 10.1038/nature13145 PMC4943525

[92] Nakashima K, Zhou X, Kunkel G, et al. The novel zinc finger-containing transcription factor osterix is required for osteoblast differentiation and bone formation. Cell 2002;108:17–29. 11792318 10.1016/s0092-8674(01)00622-5

[93] Langen UH, Pitulescu ME, Kim JM, et al. Cell-matrix signals specify bone endothelial cells during developmental osteogenesis. Nat Cell Biol 2017;19:189–201. 28218908 10.1038/ncb3476 PMC5580829

[94] Watson EC, Adams RH. Biology of bone: the vasculature of the skeletal system. Cold Spring Harb Perspect Med 2018;8:a031559. 28893838 10.1101/cshperspect.a031559 PMC6027931

[95] Burnstock G. Local mechanisms of blood flow control by perivascular nerves and endothelium. J Hypertens Suppl 1990;8:S95–106. 1982771

[96] Godo S, Shimokawa H. Endothelial functions. Arterioscler Thromb Vasc Biol 2017;37:e108–14. 28835487 10.1161/ATVBAHA.117.309813

[97] Tesfamariam B, Weisbrod RM, Cohen RA. Endothelium inhibits responses of rabbit carotid artery to adrenergic nerve stimulation. Am J Physiol 1987;253:H792–798. 3499086 10.1152/ajpheart.1987.253.4.H792

[98] Guimaraes S, Moura D. Vascular adrenoceptors: an update. Pharmacol Rev 2001;53:319–56. 11356987

[99] Luscher TF, Yang Z, Tschudi M, et al. Interaction between endothelin-1 and endothelium-derived relaxing factor in human arteries and veins. Circ Res 1990;66:1088–94. 2180587 10.1161/01.res.66.4.1088

[100] Potente M, Gerhardt H, Carmeliet P. Basic and therapeutic aspects of angiogenesis. Cell 2011;146:873–87. 21925313 10.1016/j.cell.2011.08.039

[101] Makanya AN, Hlushchuk R, Djonov VG. Intussusceptive angiogenesis and its role in vascular morphogenesis, patterning, and remodeling. Angiogenesis 2009;12:113–23. 19194777 10.1007/s10456-009-9129-5

[102] Djonov V, Baum O, Burri PH. Vascular remodeling by intussusceptive angiogenesis. Cell Tissue Res 2003;314:107–17. 14574551 10.1007/s00441-003-0784-3

[103] Blanco R, Gerhardt H. VEGF and Notch in tip and stalk cell selection. Cold Spring Harb Perspect Med 2013;3:a006569. 23085847 10.1101/cshperspect.a006569 PMC3530037

[104] Gerhardt H, Golding M, Fruttiger M, et al. VEGF guides angiogenic sprouting utilizing endothelial tip cell filopodia. J Cell Biol 2003;161:1163–77. 12810700 10.1083/jcb.200302047 PMC2172999

[105] Tymko MM, Lawley JS, Ainslie PN, et al. Global Reach 2018 Heightened alpha-Adrenergic Signaling Impairs Endothelial Function During Chronic Exposure to Hypobaric Hypoxia. Circ Res 2020;127:e1–e13. 32268833 10.1161/CIRCRESAHA.119.316053 PMC7483295

[106] Swenson ER. Sympathetic nervous system activation and vascular endothelial function with chronic hypoxia. Circ Res 2020;127:247–8. 32614721 10.1161/CIRCRESAHA.120.317114

[107] Ungvari Z, Tarantini S, Kiss T, et al. Endothelial dysfunction and angiogenesis impairment in the ageing vasculature. Nat Rev Cardiol 2018;15:555–65. 29795441 10.1038/s41569-018-0030-z PMC6612360

[108] Chen HI, Li HT, Chen CC. Physical conditioning decreases norepinephrine-induced vasoconstriction in rabbits. Possible roles of norepinephrine-evoked endothelium-derived relaxing factor. Circulation 1994;90:970–5. 8044969 10.1161/01.cir.90.2.970

[109] Kalluri R, LeBleu VS. The biology, function, and biomedical applications of exosomes. Science 2020;367:eaau6977. 32029601 10.1126/science.aau6977 PMC7717626

[110] Harding C, Heuser J, Stahl P. Receptor-mediated endocytosis of transferrin and recycling of the transferrin receptor in rat reticulocytes. J Cell Biol 1983;97:329–39. 6309857 10.1083/jcb.97.2.329 PMC2112509

[111] Pan BT, Teng K, Wu C, et al. Electron microscopic evidence for externalization of the transferrin receptor in vesicular form in sheep reticulocytes. J Cell Biol 1985;101:942–8. 2993317 10.1083/jcb.101.3.942 PMC2113705

[112] Raposo G, Nijman HW, Stoorvogel W, et al. B lymphocytes secrete antigen-presenting vesicles. J Exp Med 1996;183:1161–72. 8642258 10.1084/jem.183.3.1161 PMC2192324

[113] Huang-Doran I, Zhang CY, Vidal-Puig A, et al. Extracellular vesicles: novel mediators of cell communication in metabolic disease. Trends Endocrinol Metab 2017;28:3–18. 27810172 10.1016/j.tem.2016.10.003

[114] Gurunathan S, Kang MH, Jeyaraj M, et al. Review of the isolation, characterization, biological function, and multifarious therapeutic approaches of exosomes. Cells 2019;8:307. 30987213 10.3390/cells8040307 PMC6523673

[115] Takahashi A, Okada R, Nagao K, et al. Exosomes maintain cellular homeostasis by excreting harmful DNA from cells. Nat Commun 2017;8:15287. 28508895 10.1038/ncomms15287 PMC5440838

[116] Mathieu M, Martin-Jaular L, Lavieu G, et al. Specificities of secretion and uptake of exosomes and other extracellular vesicles for cell-to-cell communication. Nat Cell Biol 2019;21:9–17. 30602770 10.1038/s41556-018-0250-9

[117] Thery C, Zitvogel L, Amigorena S. Exosomes: composition, biogenesis and function. Nat Rev Immunol 2002;2:569–79. 12154376 10.1038/nri855

[118] Pegtel DM, Gould SJ. Exosomes. Annu Rev Biochem 2019;88:487–514. 31220978 10.1146/annurev-biochem-013118-111902

[119] Liu J, Ren L, Li S, et al. The biology, function, and applications of exosomes in cancer. Acta Pharm Sin B 2021;11:2783–97. 34589397 10.1016/j.apsb.2021.01.001 PMC8463268

[120] van Niel G, D’Angelo G, Raposo G. Shedding light on the cell biology of extracellular vesicles. Nat Rev Mol Cell Biol 2018;19:213–28. 29339798 10.1038/nrm.2017.125

[121] McAndrews KM, Kalluri R. Mechanisms associated with biogenesis of exosomes in cancer. Mol Cancer 2019;18:52. 30925917 10.1186/s12943-019-0963-9 PMC6441149

[122] Thery C, Witwer KW, Aikawa E, et al. Minimal information for studies of extracellular vesicles 2018 (MISEV2018): a position statement of the International Society for Extracellular Vesicles and update of the MISEV2014 guidelines. J Extracell Vesicles 2018;7:1535750. 30637094 10.1080/20013078.2018.1535750 PMC6322352

[123] Shah R, Patel T, Freedman JE. Circulating extracellular vesicles in human disease. N Engl J Med 2018;379:958–66. 30184457 10.1056/NEJMra1704286

[124] Alvarez-Erviti L, Seow Y, Yin H, et al. Delivery of siRNA to the mouse brain by systemic injection of targeted exosomes. Nat Biotechnol 2011;29:341–5. 21423189 10.1038/nbt.1807

[125] Kamerkar S, LeBleu VS, Sugimoto H, et al. Exosomes facilitate therapeutic targeting of oncogenic KRAS in pancreatic cancer. Nature 2017;546:498–503. 28607485 10.1038/nature22341 PMC5538883

[126] Wang Q, Yu J, Kadungure T, et al. ARMMs as a versatile platform for intracellular delivery of macromolecules. Nat Commun 2018;9:960. 29511190 10.1038/s41467-018-03390-x PMC5840177

[127] Taraboletti G, D’Ascenzo S, Borsotti P, et al. Shedding of the matrix metalloproteinases MMP-2, MMP-9, and MT1-MMP as membrane vesicle-associated components by endothelial cells. Am J Pathol 2002;160:673–80. 11839588 10.1016/S0002-9440(10)64887-0 PMC1850663

[128] Critser PJ, Yoder MC. Endothelial colony-forming cell role in neoangiogenesis and tissue repair. Curr Opin Organ Transplant 2010;15:68–72. 19898235 10.1097/MOT.0b013e32833454b5 PMC2880951

[129] Deregibus MC, Cantaluppi V, Calogero R, et al. Endothelial progenitor cell derived microvesicles activate an angiogenic program in endothelial cells by a horizontal transfer of mRNA. Blood 2007;110:2440–8. 17536014 10.1182/blood-2007-03-078709

[130] Giricz Z, Varga ZV, Baranyai T, et al. Cardioprotection by remote ischemic preconditioning of the rat heart is mediated by extracellular vesicles. J Mol Cell Cardiol 2014;68:75–8. 24440457 10.1016/j.yjmcc.2014.01.004

[131] Femmino S, Penna C, Margarita S, et al. Extracellular vesicles and cardiovascular system: biomarkers and Cardioprotective Effectors. Vascul Pharmacol 2020;135:106790. 32861822 10.1016/j.vph.2020.106790

[132] Potz BA, Scrimgeour LA, Pavlov VI, et al. Extracellular vesicle injection improves myocardial function and increases angiogenesis in a swine model of chronic ischemia. J Am Heart Assoc 2018;7:e008344. 29895586 10.1161/JAHA.117.008344 PMC6220556

[133] Cantaluppi V, Gatti S, Medica D, et al. Microvesicles derived from endothelial progenitor cells protect the kidney from ischemia-reperfusion injury by microRNA-dependent reprogramming of resident renal cells. Kidney Int 2012;82:412–27. 22495296 10.1038/ki.2012.105

[134] Zhang J, Chen C, Hu B, et al. Exosomes derived from human endothelial progenitor cells accelerate cutaneous wound healing by promoting angiogenesis through Erk1/2 signaling. Int J Biol Sci 2016;12:1472–87. 27994512 10.7150/ijbs.15514 PMC5166489

[135] Zhuang Y, Cheng M, Li M, et al. Small extracellular vesicles derived from hypoxic mesenchymal stem cells promote vascularized bone regeneration through the miR-210-3p/EFNA3/PI3K pathway. Acta Biomater 2022;150:413–26. 35850484 10.1016/j.actbio.2022.07.015

[136] Liu L, Zheng CX, Zhao N, et al. Mesenchymal stem cell aggregation-released extracellular vesicles induce CD31(+) EMCN(+) vessels in skin regeneration and improve diabetic wound healing. Adv Healthc Mater 2023;12:e2300019. 36999744 10.1002/adhm.202300019

[137] Friedenstein AJ, Chailakhyan RK, Gerasimov UV. Bone marrow osteogenic stem cells: in vitro cultivation and transplantation in diffusion chambers. Cell Tissue Kinet 1987;20:263–72. 3690622 10.1111/j.1365-2184.1987.tb01309.x

[138] Owen M, Friedenstein AJ. Stromal stem cells: marrow-derived osteogenic precursors. Ciba Found Symp 1988;136:42–60. 3068016 10.1002/9780470513637.ch4

[139] Samsonraj RM, Raghunath M, Nurcombe V, et al. Concise review: multifaceted characterization of human mesenchymal stem cells for use in regenerative medicine. Stem Cells Transl Med 2017;6:2173–85. 29076267 10.1002/sctm.17-0129 PMC5702523

[140] Timmers L, Lim SK, Arslan F, et al. Reduction of myocardial infarct size by human mesenchymal stem cell conditioned medium. Stem Cell Res 2007;1:129–37. 19383393 10.1016/j.scr.2008.02.002

[141] van Koppen A, Joles JA, van Balkom BW, et al. Human embryonic mesenchymal stem cell-derived conditioned medium rescues kidney function in rats with established chronic kidney disease. PLoS One 2012;7:e38746. 22723882 10.1371/journal.pone.0038746 PMC3378606

[142] Lai RC, Arslan F, Lee MM, et al. Exosome secreted by MSC reduces myocardial ischemia/reperfusion injury. Stem Cell Res 2010;4:214–22. 20138817 10.1016/j.scr.2009.12.003

[143] Ono M, Kosaka N, Tominaga N, et al. Exosomes from bone marrow mesenchymal stem cells contain a microRNA that promotes dormancy in metastatic breast cancer cells. Sci Signaling 2014;7:ra63. 10.1126/scisignal.2005231 24985346

[144] Phinney DG, Di Giuseppe M, Njah J, et al. Mesenchymal stem cells use extracellular vesicles to outsource mitophagy and shuttle microRNAs. Nat Commun 2015;6:8472. 26442449 10.1038/ncomms9472 PMC4598952

[145] Rani S, Ryan AE, Griffin MD, et al. Mesenchymal stem cell-derived extracellular vesicles: toward cell-free therapeutic applications. Mol Ther 2015;23:812–23. 25868399 10.1038/mt.2015.44 PMC4427881

[146] Eirin A, Zhu XY, Puranik AS, et al. Mesenchymal stem cell-derived extracellular vesicles attenuate kidney inflammation. Kidney Int 2017;92:114–24. 28242034 10.1016/j.kint.2016.12.023 PMC5483390

[147] Haga H, Yan IK, Takahashi K, et al. Extracellular vesicles from bone marrow-derived mesenchymal stem cells improve survival from lethal hepatic failure in mice. Stem Cells Transl Med 2017;6:1262–72. 28213967 10.1002/sctm.16-0226 PMC5442843

[148] Li L, Zhang Y, Mu J, et al. Transplantation of human mesenchymal stem-cell-derived exosomes immobilized in an adhesive hydrogel for effective treatment of spinal cord injury. Nano Lett 2020;20:4298–305. 32379461 10.1021/acs.nanolett.0c00929

[149] Liu W, Li L, Rong Y, et al. Hypoxic mesenchymal stem cell-derived exosomes promote bone fracture healing by the transfer of miR-126. Acta Biomater 2020;103:196–212. 31857259 10.1016/j.actbio.2019.12.020

[150] Tao SC, Yuan T, Zhang YL, et al. Exosomes derived from miR-140-5p-overexpressing human synovial mesenchymal stem cells enhance cartilage tissue regeneration and prevent osteoarthritis of the knee in a rat model. Theranostics 2017;7:180–95. 28042326 10.7150/thno.17133 PMC5196895

[151] Kishore R, Khan M. More than tiny sacks: stem cell exosomes as cell-free modality for cardiac repair. Circ Res 2016;118:330–43. 26838317 10.1161/CIRCRESAHA.115.307654 PMC4743531

[152] Haney MJ, Klyachko NL, Zhao Y, et al. Exosomes as drug delivery vehicles for Parkinson’s disease therapy. J Control Release 2015;207:18–30. 25836593 10.1016/j.jconrel.2015.03.033 PMC4430381

[153] Yu M, Liu W, Li J, et al. Exosomes derived from atorvastatin-pretreated MSC accelerate diabetic wound repair by enhancing angiogenesis via AKT/eNOS pathway. Stem Cell Res Ther 2020;11:350. 32787917 10.1186/s13287-020-01824-2 PMC7425015

[154] Fatima F, Ekstrom K, Nazarenko I, et al. Non-coding RNAs in mesenchymal stem cell-derived extracellular vesicles: deciphering regulatory roles in stem cell potency, inflammatory resolve, and tissue regeneration. Front Genet 2017;8:161. 29123544 10.3389/fgene.2017.00161 PMC5662888

[155] Teng X, Chen L, Chen W, et al. Mesenchymal stem cell-derived exosomes improve the microenvironment of infarcted myocardium contributing to angiogenesis and anti-inflammation. Cell Physiol Biochem 2015;37:2415–24. 26646808 10.1159/000438594

[156] Zhang HC, Liu XB, Huang S, et al. Microvesicles derived from human umbilical cord mesenchymal stem cells stimulated by hypoxia promote angiogenesis both *in vitro* and *in vivo*. Stem Cells Dev 2012;21:3289–97. 22839741 10.1089/scd.2012.0095 PMC3516422

[157] Zou X, Gu D, Xing X, et al. Human mesenchymal stromal cell-derived extracellular vesicles alleviate renal ischemic reperfusion injury and enhance angiogenesis in rats. Am J Transl Res 2016;8:4289–99. 27830012 PMC5095321

[158] Ma J, Zhao Y, Sun L, et al. Exosomes Derived from Akt-Modified Human Umbilical Cord Mesenchymal Stem Cells Improve Cardiac Regeneration and Promote Angiogenesis via Activating Platelet-Derived Growth Factor D. Stem Cells Transl Med 2017;6:51–9. 28170176 10.5966/sctm.2016-0038 PMC5442756

[159] Wu M, Liu X, Li Z, et al. SHED aggregate exosomes shuttled miR-26a promote angiogenesis in pulp regeneration via TGF-beta/SMAD2/3 signalling. Cell Prolif 2021;54:e13074. 34101281 10.1111/cpr.13074 PMC8249784

[160] Chen S, Tang Y, Liu Y, et al. Exosomes derived from miR-375-overexpressing human adipose mesenchymal stem cells promote bone regeneration. Cell Prolif 2019;52:e12669. 31380594 10.1111/cpr.12669 PMC6797519

[161] Li W, Liu Y, Zhang P, et al. Tissue-engineered bone immobilized with human adipose stem cells-derived exosomes promotes bone regeneration. ACS Appl Mater Interfaces 2018;10:5240–54. 29359912 10.1021/acsami.7b17620

[162] Li X, Zheng Y, Hou L, et al. Exosomes derived from maxillary BMSCs enhanced the osteogenesis in iliac BMSCs. Oral Dis 2020;26:131–44. 31541596 10.1111/odi.13202

[163] Qi X, Zhang J, Yuan H, et al. Exosomes secreted by human-induced pluripotent stem cell-derived mesenchymal stem cells repair critical-sized bone defects through enhanced angiogenesis and Osteogenesis in Osteoporotic rats. Int J Biol Sci 2016;12:836–49. 27313497 10.7150/ijbs.14809 PMC4910602

[164] Liao W, Ning Y, Xu HJ, et al. BMSC-derived exosomes carrying microRNA-122-5p promote proliferation of osteoblasts in osteonecrosis of the femoral head. Clin Sci 2019;133:1955–75. 10.1042/CS20181064 31387936

[165] Zhang L, Jiao G, Ren S, et al. Exosomes from bone marrow mesenchymal stem cells enhance fracture healing through the promotion of osteogenesis and angiogenesis in a rat model of nonunion. Stem Cell Res Ther 2020;11:38. 31992369 10.1186/s13287-020-1562-9 PMC6986095

[166] Zhang J, Liu X, Li H, et al. Exosomes/tricalcium phosphate combination scaffolds can enhance bone regeneration by activating the PI3K/Akt signaling pathway. Stem Cell Res Ther 2016;7:136. 27650895 10.1186/s13287-016-0391-3 PMC5028974

[167] Lu Z, Chen Y, Dunstan C, et al. Priming adipose stem cells with tumor necrosis factor-alpha preconditioning potentiates their exosome efficacy for bone regeneration. Tissue Eng Part A 2017;23:1212–20. 28346798 10.1089/ten.tea.2016.0548

[168] Zhu Q, Heon M, Zhao Z, et al. Microfluidic engineering of exosomes: editing cellular messages for precision therapeutics. Lab Chip 2018;18:1690–703. 29780982 10.1039/c8lc00246k PMC5997967

[169] Sun D, Zhuang X, Xiang X, et al. A novel nanoparticle drug delivery system: the anti-inflammatory activity of curcumin is enhanced when encapsulated in exosomes. Mol Ther 2010;18:1606–14. 20571541 10.1038/mt.2010.105 PMC2956928

[170] Pascucci L, Cocce V, Bonomi A, et al. Paclitaxel is incorporated by mesenchymal stromal cells and released in exosomes that inhibit in vitro tumor growth: a new approach for drug delivery. J Control Release 2014;192:262–70. 25084218 10.1016/j.jconrel.2014.07.042

[171] Kim MS, Haney MJ, Zhao Y, et al. Development of exosome-encapsulated paclitaxel to overcome MDR in cancer cells. Nanomedicine 2016;12:655–64. 26586551 10.1016/j.nano.2015.10.012 PMC4809755

[172] Fuhrmann G, Serio A, Mazo M, et al. Active loading into extracellular vesicles significantly improves the cellular uptake and photodynamic effect of porphyrins. J Control Release 2015;205:35–44. 25483424 10.1016/j.jconrel.2014.11.029

[173] Sato YT, Umezaki K, Sawada S, et al. Engineering hybrid exosomes by membrane fusion with liposomes. Sci Rep 2016;6:21933. 26911358 10.1038/srep21933 PMC4766490

